# The Intersection between Alcohol use and Sexual Activity among Young Adult Male U.S. Service Members

**DOI:** 10.2174/17450179-v19-230809-2023-9

**Published:** 2023-09-06

**Authors:** Lindsay M. Orchowski, Bryce M Meerhaeghe, Amber R. Lane, Donna M. Kazemi, Brian Borsari, Cristóbal S. Berry-Cabán

**Affiliations:** 1 Department of Psychiatry and Human Behavior Alpert Medical School of Brown University Staff Psychologist, Rhode Island Hospital, United States; 2 Department of Clinical Investigation, Womack Army Medical Center, Fort Bragg, NC, United States; 3 Department of Clinical Investigation, Womack Army Medical Center, Fort Liberty, NC, United States; 4 College of Nursing, University of South Carolina, Columbia, SC, United States; 5 San Francisco VA Health Care System, San Francisco, CA. and the Department of Psychiatry and Behavioral Sciences, UCSF Weill Institute for Neurosciences, University of California, San Francisco, CA, United States; 6 Department of Clinical Investigation, Womack Army Medical Center, Fort Bragg, NC, United States

**Keywords:** Alcohol, Sexual activity, Male soldiers, Military, MOS, Sexual coercion

## Abstract

**Aims:**

The current study explores drinking habits, preferences for alcohol use before sexual activity, and alcohol-related sexual behavior among young adult male active duty service members in the United States.

**Background:**

Hazardous alcohol use is a significant problem among United States military service members. Whereas the association between alcohol use and sexual assault is well documented in civilian samples, less is known regarding the intersection of alcohol use and sexual activity among soldiers.

**Objective:**

Descriptive statistics were utilized to summarize drinking habits, preferences for alcohol use before sexual activity, and alcohol-related sexual behavior.

**Methods:**

A sample of 338 active-duty male service members between the ages of 18 and 24 were recruited from a large military post in the Southeastern United States. Constructs were assessed using self-report surveys.

**Results:**

Participants reported consuming alcohol, on average, 5.6 times over the prior month. Average alcohol consumption was reported to be 7.8 beverages per drinking occasion. Participants reported engaging in heavy drinking an average of 2.9 times over the past 30 days. On average, service members reported a preference for 1.3 drinks before sexual activity. Furthermore, 75.2% of participants preferred to be sober during sex, and 82.1% preferred to engage in sexual activity with a sober partner. Approximately 14% of the sample reported using alcohol to improve their chances of having sex.

**Conclusion:**

These findings highlight high rates of alcohol use among soldiers. Nonetheless, young adult male soldiers report a preference for sexual activity while sober. Understanding the co-occurrence of alcohol use and sexual activity has the potential to inform the development of integrated alcohol and sexual assault prevention programs for service members.

## 1 INTRODUCTION

Hazardous alcohol use is a significant concern for the United States military [1]. Approximately 30% of active duty service members engage in binge drinking [2], defined as consuming five or more drinks on the same occasion for men and consuming four or more drinks on the same occasion for women [3]. Sexual violence is also a serious concern for the military [4]. A large survey of U.S. service members found that 4.9% of women and 1% of men experienced past year sexual assault [5]. According to the 2018 Workplace Gender Relations Assessment among service members, alcohol was consumed by the victim and/or perpetrator among 62% of women and 49% of men who reported past year sexual assault [6].

Studies among civilians suggest alcohol use is a risk factor for sexual aggression [7]. When drinking, men misinterpret friendly cues as a sign of sexual interest [8]. Environmental factors – such as party or bar attendance – are also predictive of sexual assault perpetration, with the association between drinking settings and perpetration explained by higher engagement in casual sex (*i.e*., “hookups”) [9]. Deliberate administration of alcohol use to engage a partner in sexual activity is also recognized as a form of sexual coercion [10].

Despite a strong link between alcohol and sexual assault among civilians, relatively little is known regarding the intersection of alcohol and risky sexual activity among soldiers. The current research, therefore, sought to describe alcohol use, preferences for using alcohol prior to sexual activity, and engagement in alcohol-related sexual behavior (*i.e*., using alcohol prior to sexual activity, deliberately giving a woman alcohol to increase the chances of sex) among male soldiers. Given that sexual assault prevention programs are beginning to focus on the intersection of alcohol use and sexual assault among civilians [11, 12], these data can inform the development of integrated alcohol and sexual assault prevention efforts for the military.

## 2 MATERIALS AND METHODS

### 2.1 Participants

To be eligible for the current study, participants needed to be men between the ages of 18 to 24 and be engaged in active-duty military service. The study sample included 338 male service members stationed at a large Southeastern Army installation in the United States. Most participants were between the ages of 20 and 24 (88.8%; mean age = 21.6 years). Regarding race and ethnicity, 50.6% self-identified as white, 18.0% as black, and 23.6% as Hispanic. Regarding marital status, most participants indicated that they were not married (68.4%). Over one-third (37.1%) were serving in a combat role. Regarding sexual attraction, 90.9%) indicated being only attracted to women.

### 2.2 Measures

#### 2.2.1 Demographics

Participants completed items assessing age, race/ethnicity, relationship status, sexual orientation, and military occupational specialty (MOS), which is a code used by the US Army to identify specific job categories.

#### 2.2.2 Alcohol Use

Participants indicated the number of occasions they consumed alcohol in the past month, the highest number of drinks consumed in one sitting, and the number of occasions in which they consumed five or more drinks in one sitting. The survey provided participants with the definition of a standard drink. Participants were also asked to self-identify in one of five categories based on their alcohol use habits. “Regular drinker” was defined as typically consuming one or more drinks per month; “occasional drinkers” consumed alcohol at least once in the past year; and “infrequent drinkers” consumed alcohol previously but have not done so in the past year. Participants were also provided the option of stating they never consumed alcohol or were “lifelong non-drinkers.”

#### 2.2.3 Alcohol Use and Sexual Experiences

Participants reported their preferred number of drinks for themselves and their partners before engaging in sexual intercourse. Using colloquial terms, participants were asked to disclose their preferred level of intoxication themselves, including: “sober,” “buzzed,” “drunk,” and “wasted.”

#### 2.2.4 Alcohol-related Sexual Behavior

Participants completed four questions to ascertain whether they engaged in alcohol-related sexual behaviors (*i.e*., using alcohol prior to sexual activity, deliberately giving a woman alcohol to increase the chances of sexual activity). Participants responded “yes” or “no” to the following prompts: 1) Have you ever given alcohol to a woman or encouraged her to drink in order to increase the chances of having sex (*e.g.*, buying alcohol for a woman, providing alcohol for a woman, suggesting that the woman drink)?; 2) Did you use alcohol the last time you had intercourse with a long term partner?; 3) Did you use alcohol the last time you had intercourse with someone you recently met?; 4) Did you use alcohol the last time you had intercourse in a paid exchange?

### 2.3 Procedure

Recruitment for the study was conducted by a team of trained male research assistants. The study was advertised as a survey of alcohol use and social behavior. Because participants were compensated with a $10 gift card for their time completing the survey, only service members who indicated that they were currently off-duty were eligible to participate. Participants completed informed consent *via* an electronic consent statement. The survey took approximately 15 minutes to complete and was administered on an iPad tablet computer. The survey was anonymous, and no identifying information was collected. The study was approved by the Defense Health Agency Regional Health Command-Atlantic Institutional Review Board (RHC-A-18-011).

## 3 RESULTS

### 3.1 Alcohol Use

Respondents consumed alcohol an average of 5.6 times in the past 30 days (*SD* = 8.6). Participants also reported drinking an average of 7.8 alcoholic beverages as the highest amount consumed in one sitting (*SD* = 16.9) and engaged in heavy episodic drinking an average of 2.9 times during the past 30 days (*SD* = 5.2) (Table **1**). Further, 26.2% of those in a combat specialty self-identified as regular drinkers (Table **2**).

### 3.2 Preferences Regarding Alcohol Use and Sexual Activity

Participants reported a preference for consuming an average of 1.3 drinks prior to sexual activity (*SD* = 3.0) and preferred sexual activity with a partner that had consumed less than a full drink (*M* = 0.7, *SD* = 2.7; Table **2**). Most men (75.3%) preferred to be sober during sexual activity and indicated a preference for their partner to be sober (82.1%) (Table **3**).

### 3.3 Engagement in Alcohol-Related Sexual Activity

Approximately one-quarter of participants (25.7%) used alcohol prior to sex with a long-term partner, and 13.6% reported using alcohol prior to sexual activity with a new partner. Furthermore, 13.6% reported using alcohol to increase their chances of having sex, and 1.4% reported using alcohol during a paid exchange for sex (Table **4**).

## 4 DISCUSSION

This study examines the intersection of alcohol use and sexual activity among young adult men in active-duty military service. Participants’ drinking habits in the past 30 days were consistent with other studies among service members [2]. Approximately 1 in 4 men (26.2%) in combat specialties identified as regular drinkers. This may be because individuals serving in combat-related specialties report more severe psychological effects associated with their position, which may increase the likelihood of habitual alcohol use [13, 14].

Approximately 1 in 4 men reported engaging in alcohol use prior to sexual activity with a consistent partner, and 1 in 7 men engaged in alcohol use prior to sexual activity with a new partner. These data mirror rates of alcohol-involved sex reported in studies of civilians. For example, Goldstein *et al.* [15] found that 26% of sexually active college students reported alcohol-related sexual behavior with someone they had met in the past 3-months. Most service members also reported a preference to be sober during sexual activity. These data are positive, given that alcohol use prior to or during sexual activity is associated with riskier sex [16].

These findings also have implications for sexual assault prevention. A preference for abstaining from alcohol use prior to sexual activity contradicts the cultural belief that alcohol is an essential component of sexual encounters [17, 18]. There are a growing number of sexual assault prevention programs that draw upon social norms theory to correct misperceptions of peer engagement in risk behavior [19, 12]. The current data can inform sexual assault prevention efforts for service members who take such an approach. For example, given that the majority of soldiers did not consume alcohol prior to sex, this information can be utilized to reinforce the norm that most soldiers engage in sexual activity when sober.

Notably, 14% of service members reported engaging in sexual coercion by giving alcohol to a woman or encouraging her to drink to increase the chances of having sex. The Revised Sexual Experiences Survey [10] recognizes taking advantage of a woman when they were too drunk or out of it to smiddle what was happening as a form of sexual aggression. Deliberate administration of alcohol is a common tactic used to perpetrate sexual aggression among civilians [20]. The current data therefore provide some insight into the prevalence of this form of sexual coercion among young adult male soldiers in the US.

## CONCLUSION

Several limitations should be noted. Although the survey was anonymous, participants may have been influenced by social desirability biases. Given that sexual aggression is often perpetrated by men, participants in this study were limited to young adult male service members. Future research should include women as well as service members of other ages. Colloquial terms were utilized to describe their preferred level of intoxication. No standard definition of these terms was provided to participants, and their understanding of these terms may have varied as a result. The sample may also not be generalizable, and findings may be influenced by factors such as culture and religion. Despite these limitations, this research adds to the literature by describing the intersection of alcohol use and sexual activity among soldiers. It builds upon existing research examining the intersection of alcohol use and sexual activity among US military personnel [21]. Data suggesting that 1 in 7 men used alcohol to increase their chances of sexual activity highlight the need to target alcohol in sexual assault prevention. Data suggesting that most men preferred sexual activity while sober can also be utilized to correct misperceived norms regarding the co-occurrence of alcohol and sex.

## Figures and Tables

**Table 1:** Drinking behavior and preference for drinking before sexual activity.

**Item**	**n**	**M**	**SD**
Occasions in the past 30 days	313	5.64	8.63
Number of times consuming five or more drinks in one sitting in the past 30 days	316	2.89	5.12
The highest number of drinks in one sitting in the past 30 days	314	7.84	16.78
Preferred number of drinks before sex, self	307	1.26	2.96
Preferred number of drinks before sex, partner	311	0.68	2.73

**Table 2:** Drinking patterns.

Drinking Classifications										
-	Regular Drinker		Occasional Drinker		Infrequent Drinker		Never Consumed		Non-Drinker	
-	*n*	*%*	*n*	*%*	*n*	*%*	*n*	*%*	*n*	*%*
Race (*n* = 308)	-	-	-	-	-	-	-	-	-
White	91	29.5	30	9.7	14	4.5	14	4.5	5	1.6
Black	28	9.1	12	3.9	5	1.6	8	2.6	2	0.6
Hispanic	42	13.6	16	5.2	6	1.9	7	2.3	3	1.0
Asian	6	1.9	2	0.6	1	0.3	0	0.0	0	0.0
Other	10	3.2	6	1.9	0	0.0	0	0.0	0	0.0
Age (*n* = 324)	-	-	-	-	-	-	-	-	-	-
18	3	9.3	1	0.3	0	0.0	0	0.0	0	0.0
19	15	4.6	5	1.5	4	1.2	8	2.5	0	0.0
20	30	9.6	12	3.7	4	1.2	15	4.6	5	1.5
21	45	13.9	11	3.4	4	1.2	2	0.6	1	0.3
22	28	8.6	10	3.1	5	1.5	3	0.9	2	0.6
23	25	7.7	14	4.3	7	2.2	1	0.3	2	0.6
24	38	11.7	16	4.9	4	1.2	3	0.9	1	0.3
MOS (*n* = 286)	-	-	-	-	-	-	-	-	-
Combat	75	26.2	15	5.2	6	2.1	11	3.8	1	0.3
Maintenance	31	10.8	13	4.5	6	2.1	9	3.1	4	1.4
Medic	19	6.6	7	2.4	1	0.3	1	0.3	0	0.0
Construction	14	4.9	3	1.0	4	1.4	0	0.0	2	0.7
Communications	8	2.8	3	1.0	2	0.7	0	0.0	3	1.0
Other	22	7.7	14	4.9	7	2.4	4	1.4	1	0.3

**Table 3:** Preference for alcohol and sexual activity.

-		***n***	***%***
Self (*n* = 319)	Sober	240	75.2
	Buzzed	68	21.3
	Drunk	7	2.2
	Wasted	4	1.3
Partner (*n* = 318)	Sober	261	82.1
	Buzzed	51	16.0
	Drunk	4	1.3
	Wasted	2	0.6

**Table 4:** Alcohol use and risky sexual behavior.

-	**Total Responding** **“Yes”**	
-	***n***	***%***
Alcohol Use During Sex with a Long-Term Partner (*n* = 311)	80	25.7
Alcohol Use During Sex with a New Partner (*n* = 313)	43	13.6
Used Alcohol to Increase Chances of Having Sex (*n* = 317)	43	13.6
Alcohol in a Paid Exchange for Sex (*n* = 312)	4	1.4

## Data Availability

The data supporting the findings of the article is available by request from the primary author, [L.O].

## LIST OF ABBREVIATIONS

MOS: = Military Occupational Specialty
US: = United States
HRBS: = Health Related Behaviors Survey
